# A comparison of uni- and multi-variate methods for identifying brain networks activated by cognitive tasks using intracranial EEG

**DOI:** 10.3389/fnins.2022.946240

**Published:** 2022-09-26

**Authors:** Cristian Donos, Bogdan Blidarescu, Constantin Pistol, Irina Oane, Ioana Mindruta, Andrei Barborica

**Affiliations:** ^1^Department of Physics, University of Bucharest, Bucharest, Romania; ^2^Epilepsy Monitoring Unit, Department of Neurology, Emergency University Hospital Bucharest, Bucharest, Romania

**Keywords:** intracranial EEG (iEEG), brain network, searchlight analysis, multivariate pattern analysis (MVPA), facial emotion recognition (FER), machine learning

## Abstract

Cognitive tasks are commonly used to identify brain networks involved in the underlying cognitive process. However, inferring the brain networks from intracranial EEG data presents several challenges related to the sparse spatial sampling of the brain and the high variability of the EEG trace due to concurrent brain processes. In this manuscript, we use a well-known facial emotion recognition task to compare three different ways of analyzing the contrasts between task conditions: permutation cluster tests, machine learning (ML) classifiers, and a searchlight implementation of multivariate pattern analysis (MVPA) for intracranial sparse data recorded from 13 patients undergoing presurgical evaluation for drug-resistant epilepsy. Using all three methods, we aim at highlighting the brain structures with significant contrast between conditions. In the absence of ground truth, we use the scientific literature to validate our results. The comparison of the three methods’ results shows moderate agreement, measured by the Jaccard coefficient, between the permutation cluster tests and the machine learning [0.33 and 0.52 for the left (LH) and right (RH) hemispheres], and 0.44 and 0.37 for the LH and RH between the permutation cluster tests and MVPA. The agreement between ML and MVPA is higher: 0.65 for the LH and 0.62 for the RH. To put these results in context, we performed a brief review of the literature and we discuss how each brain structure’s involvement in the facial emotion recognition task.

## Highlights

- Intracranial EEG recordings during a facial emotion recognition task.
- The first implementation of searchlight MVPA with sparse intracranial electrodes.
- Comparison of permutation cluster tests, ML classification, and searchlight MVPA.

## Introduction

Our understanding of how the human brain works is currently at its peak, driven by significant technological and methodological advances. The 19th century marked the transition from non-scientific, and often superstitious approaches to treating and diagnosing brain-related diseases, to evidence-driven scientific research. The pioneering work of Paul Brocca on aphasia patients resulted in the first evidence of a region in the left frontal cortex being involved in the articulation of speech, and hence supported the hypothesis of localization of brain functions (Finger, 2004). Following the same reasoning, the lesion studies led to many brain functions being linked to specific brain regions (Rorden et al., 2007; Karnath et al., 2018; Vaidya et al., 2019), resulting in what we currently know as the “localizationist view.” However, a “holistic view,” that argues that brain functions are widely distributed across the cortex, emerged gained traction in the last couple of decades, fueled by the advances in technology (fMRI, MEG, etc.) and numerical methods (multivariate pattern analysis, machine learning based decoders, etc.) (Shehzad and McCarthy, 2018; Vaidya et al., 2019).

A study on brain connectivity and the networks involved in natural vision (Di and Biswal, 2020) has shown that the intersubject variability of the brain connectivity is low for the visual areas and the default mode network, but significantly higher for widespread brain networks that include brain regions that participate in the realization of a large number of functions, like the prefrontal cortex (Miller and Cohen, 2001; Lara and Wallis, 2015; Funahashi, 2017) and the anterior temporal lobe (Tsapkini et al., 2011; Rice et al., 2015). When attempting to identify the brain networks involved in a specific cognitive task, despite using the same raw data for analysis, the analysis itself may aim to highlight correlations between the task and the activation of various brain structures in an exploratory fashion (Price, 2010; Woolnough et al., 2020), or may aim to add a predictive dimension to the analysis, like in most brain-computer interface (BCI) applications (Nicolas-Alonso and Gomez-Gil, 2012; Saha et al., 2021), to generalize and use the observations to accurately predict an outcome in prospective subjects. One of the most common misconceptions is that the exploratory analysis, if performed rigorously in a well-controlled experimental setup, can be used to make predictions on prospective subjects. However, the *p*-values associated with various variables of interest identified in the exploratory analysis do not measure the predictive accuracy of the model, but merely the contribution of that variable to the realization of an outcome at a certain chance level (Bzdok and Ioannidis, 2019). Moreover, the methods relying on *p*-values are not suitable when there are multiple strategies to perform a certain cognitive task and the strategies are all represented in the study cohort. For example, to perform the cumulative sum of *n* integers, one may perform a serial summation to reach the results, may apply the formula $\frac{n\,\left(n+1\right)}{2}$, or, if n is small enough, may rely on mental imagery to calculate the result (Seghier and Price, 2018).

In this manuscript, we aim at identifying the brain areas that exhibit differential activation during a cognitive task. We chose a facial emotion recognition task, for which multiple theoretical models (Pessoa and Adolphs, 2010; You and Li, 2016; LeDoux and Brown, 2017) and significant evidence of different brain structures’ participation in the task already exist (Guillory and Bujarski, 2014), thus making it easier for us to interpret the results. At the same time, recent studies (Wager et al., 2015; Kragel and LaBar, 2016) have shown that the emotional brain response is widespread across the cortex, and requires a complex pattern of activation to identify a type of emotion. In our study, we chose representative methods for both exploratory and predictive types of analyses. We use three different methods for identifying condition contrasts: (1) a permutation cluster test, which is a commonly used technique for the analysis of EEG data (Maris and Oostenveld, 2007) in an exploratory fashion, (2) a machine learning (ML) classifier (Graimann et al., 2003) which was successfully used for epileptic seizure type prediction in prospective subjects (Donos et al., 2018) and in BCI applications (Steyrl et al., 2016), and (3) a searchlight multivariate pattern analysis (MVPA) (Haxby et al., 2001) which is a method that was successfully used in fMRI studies for identifying brain regions participating in a cognitive task in both exploratory (Kriegeskorte et al., 2006) and predictive type of analyses (Mitchell et al., 2008).

Although we will discuss the results of the three methods in the context of the existing literature on facial emotion recognition (Pessoa and Adolphs, 2010; Guillory and Bujarski, 2014), this manuscript does not aim to study the cognitive processes behind the facial emotion recognition task, but rather to highlight which analytical method is better suited for doing so. To support our findings, we present a brief review of the literature, showing that the brain regions deemed important for the facial emotion recognition task were indeed reported by other studies as well, in the context of emotion processing or supporting or co-occurring functions, such as the working memory or inner speech (Miendlarzewska et al., 2013).

## Materials and methods

Thirteen subjects undergoing stereo-EEG (SEEG) presurgical evaluation for drug-resistant epilepsy at the Bucharest University Hospital were recruited for this study. All subjects provided informed consent and the investigation was performed under the Ethical Committee approval 43/02.10.2019. All subjects were implanted with Dixi depth electrodes (Dixi, Chaudefontaine, France) having 8–18 contacts per electrode, 2 mm contact length, 3.5 mm contact spacing, and 0.8 mm diameter. The location of depth electrodes was chosen solely based on the clinical hypothesis for the epileptogenic focus. At the group level, 7 subjects had electrodes implanted in the left hemisphere, 2 subjects had electrodes implanted in the right hemisphere, and 4 subjects had bilateral implantations. The depth electrodes and their contacts were precisely localized using post-operative CT images registered on top 1.5T or 3T presurgical T1 MRIs. The presurgical MRI was also used for brain segmentation (Dale et al., 1999), parcellation (Desikan et al., 2006; Destrieux et al., 2010), and non-linear registration (Postelnicu et al., 2009) to the “cvs_avg35_inMNI152” brain template, which is available in Freesurfer. Each electrode contact was assigned to a voxel whose 3D coordinates were further used to represent it on the group template. The anatomical label, according to the Desikan–Killiany atlas (Desikan et al., 2006), of each electrode contact was chosen as the label with the largest number of voxels in a 3×3×3 cube centered on the electrode contact. We used this procedure to minimize the chance of mislabeling contacts due to noise in the MRI image or contacts lying at the border of one or more brain structures. An average of 11.31 ± 2.75 depth electrodes were implanted per subject, sampling on average 10.55 ± 5.52 left hemisphere (LH) and 17.67 ± 4.41 right hemisphere (RH) brain structures. The average number of depth electrode contacts implanted per subject was 90.64 ± 59.15 in the LH, and 167.17 ± 25.76 in the RH (Table 1).

**TABLE 1:** Patient cohort.

Subject	Sex	LH electrodes	LH structures	LH contacts	RH electrodes	RH structures	RH contacts	No contacts recorded from
SEEG85	M	YES	4	15	YES	23	192	96
SEEG88	M	YES	15	112	NO	−	−	58
SEEG89	M	YES	3	8	YES	23	164	91
SEEG90	M	YES	9	88	NO	−	−	42
SEEG92	M	YES	18	159	NO	−	−	51
SEEG94	M	YES	14	116	NO	−	−	77
SEEG96	M	NO	−	−	YES	14	158	67
SEEG97	F	YES	13	125	NO	−	−	83
SEEG98	F	NO	−	−	YES	14	138	68
SEEG99	M	YES	13	167	NO	−	−	86
SEEG101	M	YES	5	36	YES	14	147	96
SEEG102	F	YES	5	28	YES	18	204	97
SEEG104	F	YES	17	143	NO	−	−	92
TOTAL				997			1003	1004
MEAN			10.55	90.64		17.67	167.17	77.23
STD			5.52	59.15		4.41	25.79	18.49

**FIGURE 1:**
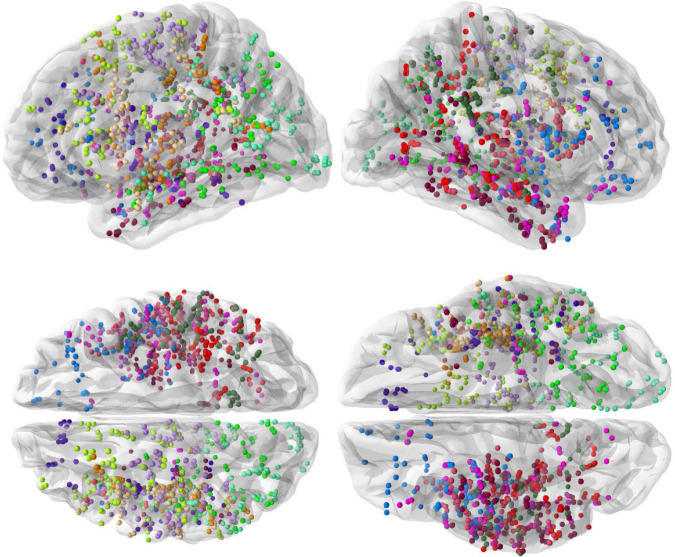
Spatial coverage of the brain by intracranial electrodes (**top row:** left and right hemisphere views, **bottom row:** dorsal and ventral views). Intracranial electrode contacts of each of the 13 subjects are shown with a different color.

The spatial coverage of the brain with depth electrodes, represented on the “fsaverage” brain template at the group level, is shown in Figure 1.

### Cognitive task

**FIGURE 2:**
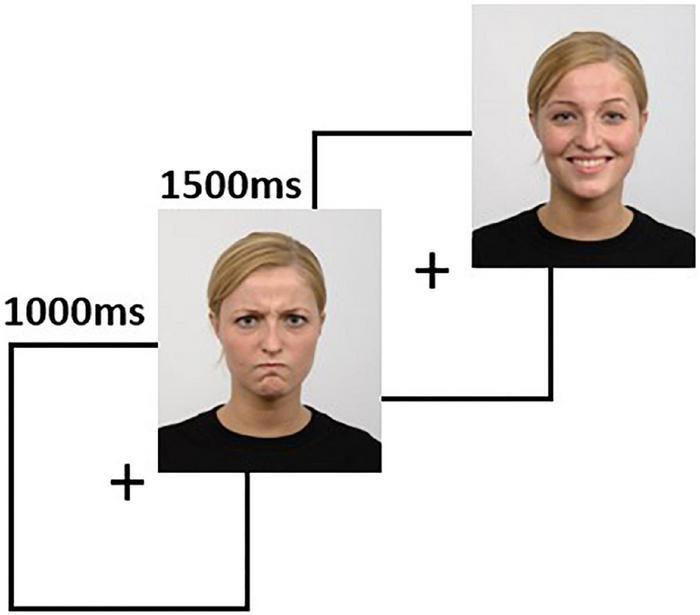
The facial emotion recognition task is comprised of sequences of 1000 ms fixation cross, followed by 1500 ms of a face image. In this figure, we show two sample trials. Source for the images in this figure: Radboud Faces Database.

The task was developed using the stimuli available in the Radboud Faces Database (Langner et al., 2010). The stimuli are pictures of 67 actors posing with neutral, negative (angry), and positive (happy) faces, with matched facial landmarks used to recreate the facial expressions. The task contains 67 trials for each condition, each trial consisting of a 1-s fixation cross, followed by 1.5 s of a face image (Figure 2). The subjects were instructed to press one of three predefined keyboard buttons to indicate if the face has a neutral, negative, or positive expression. The task presentation was accomplished using PsychoPy (Peirce et al., 2019) and a 24-inch LCD monitor placed at 114 centimeters from the subject. A photodiode was used to synchronize the rendering of the visual stimuli with the EEG recording system.

### Intracranial EEG recordings

Intracranial EEG recordings was recorded while the subjects performed the cognitive task from 93.69 ± 15.26 contacts per subject, on average. The iEEG was recorded with a sampling rate of 4096 Hz using a 128- or 256-channel XLTek Quantum Amplifiers (Natus Neuro, Middleton, WI, USA).

The iEEG processing pipeline contains a mix of open-source software and in-house code developed in Matlab and Python. The raw recordings in XLTek format were loaded in Matlab and exported in ADES format and loaded in Anywave (Colombet et al., 2015) for visual inspection and marking for removal of individual trials or EEG channels exhibiting epileptic activity or non-physiological artifacts. The remaining pipeline steps were all performed in Python, using MNE (Gramfort et al., 2013) as the main framework for creating our custom processing steps. The EEG was notch filtered at 50Hz and its second (100 Hz) and third (150 Hz) harmonics, using a finite impulse response filter with Hamming window. A common-average reference was computed using the good EEG channels, then the EEG was cropped into epochs of [−0.3; 1] s relative to the stimuli onset and resampled to 256 Hz. An additional buffer of 1 s was added for each epoch to mitigate filtering artifacts that are expected to occur at the edges of the epochs during the time-frequency transformation which is necessary to extract the gamma-band spectral content of the signal. The time-frequency decomposition was accomplished with Morlet wavelets having a variable number of cycles for each frequency in the [1; 125] Hz range. The time-frequency representation of individual trials was cropped to [−0.3; 1] s to remove filtering artifacts and was baseline corrected. The power in the gamma band was computed by averaging the power of each frequency in the [55; 115] Hz range. These gamma power traces (GPTs) represent the inputs for all subsequent analysis methods, which we describe in detail below.

### Permutation cluster analysis for task contrasts

For each EEG channel, we identified significant differences between the angry and happy task conditions in the GPTs using a non-parametric cluster-level statistical permutation test (Maris and Oostenveld, 2007). The permutation cluster test was implemented using 1,000 permutations. To assess the participation of different brain structures in the realization of the task, we aggregated at the group level the trials from all contacts within the same brain structure, and we performed the permutation cluster test as described previously. Only clusters showing significant differences between task conditions at *p*-values below 0.05 were further considered.

### Machine learning classification for task conditions

Machine learning (ML) classification for task conditions takes the idea of identifying contrasts one step further, in the sense that once an ML model is trained, it can identify the task condition of new trials. This is a very strong outcome, as it proves that the underlying neuronal population that produces the iEEG recorded by a single contact has specific responses for different conditions, and the effect size is large enough to be identified at a single-trial level.

The ML classification was implemented using the “pipeline” feature of “scikit-learn” (Buitinck et al., 2013), which allows training of a model with cross-validation (CV) while performing data augmentation and transformation and separately for each fold.

In the data augmentation step, we compute the average GPT for the angry and happy conditions, then for each trial, we create two new time series by subtracting the condition averages. Next, during the data transformation step, we split the time series into three intervals [0.2; 0.4], [0.4; 0.6], and [0.6; 1] s, and for each interval, we compute 5 statistical measures: mean, standard deviation, median, skewness, and kurtosis. The data augmentation and transformation steps are encapsulated into a Transformer interface (Buitinck et al., 2013) so that they can be re-fitted and re-applied separately to each fold of the CV without information leakage. The transformed data is fed into a random forest classifier (Breiman, 2001), whose parameters are obtained through hyperoptimization. The hyperoptimization is performed using Bayesian Search with 10-fold stratified cross-validation (Snoek et al., 2012), as implemented by the “BayesSearchCV” function in “scikit-optimize” (Head et al., 2021). The parameters we optimized for are the number of estimators (*n*_*estimators*_ ∈ {250, 500, 1000}), maximum tree depth (integer values in the range [2; 10]), and the percentage of features randomly chosen for growing each individual tree (*p*_*features*_ ∈ {0.15;0.5;0.75}). The optimizer performed 100 iterations using 6 out of 8 CPU cores of a 3.40 Ghz Intel Core i7-6700 CPU with 16 GB RAM. Once the best parameters for the random forest classifier were identified, we used a 10-fold stratified CV to evaluate the classification performance. The evaluation metric of choice was the normalized Matthews Correlation Coefficient (NMCC) (Chicco and Jurman, 2020):


$$N\,M\,C\,C=\frac{M\,C\,C+1}{2}$$

$w\,h\,e\,r\,e\,M\,C\,C=\frac{T\,N\cdot T\,N-F\,P\cdot F\,N}{\sqrt{\left(T\,P+F\,P\right)\cdot \left(T\,P+F\,N\right)\cdot \left(T\,N+F\,P\right)\cdot \left(T\,N+F\,N\right)}};$ TP = true positive; TN = true negatives; FP = false positives; FN = false negatives.

An NMCC value of 0.5 represents the random chance level, and to assess if the performance of our classifier is significantly better than chance, we employed a *t*-test against the null hypothesis that the mean of the 10 NMCC values resulting during the CV is 0.5.

The average training and evaluation time per intracranial contact was 406.8 ± 63.2 s.

### Searchlight decoding for task conditions

The multivariate searchlight was initially developed for fMRI data, for localizing functional brain regions that are informative for brain processes (Kriegeskorte et al., 2006) triggered by a cognitive task. Searchlight maps the information over a set of neighboring voxels in fMRI, within a predefined spherical volume, therefore mitigating the issues related to the multiple comparison problem using a spatial smoothing approach. In this study, we apply the same reasoning to iEEG data. A searchlight radius of 25 mm was chosen. While there is no rule of thumb for choosing the searchlight radius size, previous studies (Wang et al., 2020) have shown that increasing the search radius results in the same clusters being identified, but yielding larger cluster sizes. This behavior is welcome given the sparsity of the intracranial electrodes and the distance of 3.5 mm between two adjacent electrode contacts from the same depth electrode. For each electrode contact, we identified all electrode contacts that were within the searchlight volume defined by the searchlight radius, regardless if they were located on the same or different depth electrodes, and we used MVPA to decode the task conditions from the gamma traces (King and Dehaene, 2014; King et al., 2018). The MVPA pipeline took as input the gamma power traces for both task conditions and the condition labels associated with the gamma traces, then performed scaling, concatenation across the channel dimension, and classification using logistic regression. Therefore, the MVPA decoder aggregated information across time and space. The MVPA decoder was trained with 10-fold cross-validation, evaluated by the NMCC, and assessed significance above or below the chance level using a *t*-test, as described in the ML classification paragraph.

### Comparison of the three methods

The results of the three methods for identifying task contrasts were compared at the group level. For each method, we identified the brain structures that contained at least one contact with a significant contrast between the two conditions. We used the Jaccard index (Jaccard, 1901) to quantify the agreement between every pair of methods.

## Results

### Task performance

The average response time was 0.97 ± 0.08 s and 0.85 ± 0.11 s for the angry and happy conditions, respectively (Table 2). The average number of trials per subject, without interictal epileptic spikes and other non-physiological artifacts, and for which the subjects correctly identified the emotion was 51.54 ± 7.77 and 54.62 ± 11.38 for the angry and happy conditions. All subjects took a longer time to correctly identify the angry condition. For 10 out of 13 subjects, the difference in reaction time between the angry and happy conditions was significant (Mann–Whitney’s *U*-test, *p* < 0.05). At the group level, the differences in reaction times were significantly different as well (Mann–Whitney’s *U*-test, *p* < 0.05) (Table 2).

**TABLE 2:** Task performance.

Subject	Angry trials	Happy trials	Angry RT	Happy RT	Sig RT difference
SEEG85	45	21	0.92	0.62	0.0000
SEEG88	62	66	0.96	0.84	0.0000
SEEG89	47	58	0.97	0.85	0.0000
SEEG90	56	60	0.91	0.83	0.0117
SEEG92	53	55	0.95	0.90	0.1700
SEEG94	59	61	0.92	0.87	0.0903
SEEG96	45	54	0.97	0.75	0.0000
SEEG97	56	63	0.98	0.88	0.0005
SEEG98	58	57	0.90	0.88	0.3135
SEEG99	59	61	0.89	0.85	0.2466
SEEG101	43	53	1.05	0.84	0.0000
SEEG102	36	45	1.19	1.10	0.0216
SEEG104	51	56	0.97	0.81	0.0000
TOTAL	670	710			
MEAN	51.54	54.62	0.97	0.85	0.0003
STD	7.77	11.38	0.08	0.11	

### Brain sampling

Thirty-two brain structures were sampled by depth electrodes at the group level (32 LH and 31 RH), and a total of 1,004 electrode contacts (531 LH and 473 RH) were free of physiological and non-physiological artifacts and used in the analysis. The exact distribution of electrode contacts per implanted structure is detailed in Table 3.

**TABLE 3:** Per structure distribution of implanted electrode contacts, and the number of significant contacts per each analysis method.

Brain structure	Hemi	Implanted	Permutation	ML	MVPA	Relation to the task
AMYGDALA	LH	2	2		1	Part of the emotion processing network (Pessoa and Adolphs, 2010; LeDoux and Brown, 2017)
AMYGDALA	RH	12	12	1	5	
BANKSSTS	LH	10	10	3	5	Face processing (Hein and Knight, 2008)
BANKSSTS	RH	15	15	3	7	
CAUDALANTERIORCINGULATE	RH	3				Emotion processing (Etkin et al., 2011)
CAUDALMIDDLEFRONTAL	LH	22	22	1	3	Emotion processing (Etkin et al., 2011)
CAUDALMIDDLEFRONTAL	RH	8	8	2	4	
ENTORHINAL	RH	4	4			−
FUSIFORM	LH	25	25	3	13	Part of the visual system, specialized in facial recognition (Ganel et al., 2005; Kanwisher and Yovel, 2006; Vuilleumier and Pourtois, 2007)
FUSIFORM	RH	25	25	4	2	
HIPPOCAMPUS	LH	8			3	Encoding and recognition of facial and emotional expressions (Fried et al., 1997)
HIPPOCAMPUS	RH	35	35	2	4	
INFERIORPARIETAL	LH	17	17	1	14	Involved in decoding facial expressions (Sarkheil et al., 2013) and emotion perception (Engelen et al., 2015)
INFERIORPARIETAL	RH	20		2	13	
INFERIORTEMPORAL	LH	23	23	2	14	Part of the emotion processing network (Pessoa and Adolphs, 2010)
INFERIORTEMPORAL	RH	39	39	7	4	
INSULA	LH	57		9	3	Emotion regulation (Blair et al., 2007; Pessoa and Adolphs, 2010)
INSULA	RH	36		2	5	
ISTHMUSCINGULATE	LH	8		1	1	If damaged, may lead to dysfunctional emotional control (Wei et al., 2021)
ISTHMUSCINGULATE	RH	7		1	2	
LATERALOCCIPITAL	LH	6			6	Involved in face perception (Nagy et al., 2012)
LATERALOCCIPITAL	RH	8	8	1	2	
LATERALORBITOFRONTAL	LH	2	2			Involved in processing emotional valence (Pessoa and Adolphs, 2010; Kragel and LaBar, 2016)
LATERALORBITOFRONTAL	RH	7				
LINGUAL	LH	12		3	7	Sensitive to emotional valence (Lima Portugal et al., 2020), may cause prosopagnosia if damaged (Kesserwani and Kesserwani, 2020)
LINGUAL	RH	7			1	
MEDIALORBITOFRONTAL	LH	3		1		Involved in processing emotional valence (Pessoa and Adolphs, 2010; Kragel and LaBar, 2016)
MEDIALORBITOFRONTAL	RH	1				
MIDDLETEMPORAL	LH	35	35	4	10	Part of the emotion processing network (Pessoa and Adolphs, 2010)
MIDDLETEMPORAL	RH	72		5	15	
PARACENTRAL	LH	7		3	2	Tied to mood disorders (Zhang et al., 2021) and the extraction of social information from faces (Sarkheil et al., 2013)
PARAHIPPOCAMPAL	LH	4				Involved in emotional memory retrieval (Smith et al., 2004)
PARAHIPPOCAMPAL	RH	3			1	
PARSOPERCULARIS	LH	30		4	4	Part of ventrolateral prefrontal cortex, involved in emotion processing (Pessoa and Adolphs, 2010; Nejati et al., 2021)
PARSOPERCULARIS	RH	18	18	4		
PARSORBITALIS	RH	6		1		Involved in emotional processing (Belyk et al., 2017; Nejati et al., 2021)
PARSTRIANGULARIS	LH	6	6	2		Part of ventrolateral prefrontal cortex, involved in emotion processing (Pessoa and Adolphs, 2010; Nejati et al., 2021)
PARSTRIANGULARIS	RH	4		1		
POSTCENTRAL	LH	35		4	12	Involved in emotion recognition and regulation (Adolphs et al., 2000; Kropf et al., 2019)
POSTCENTRAL	RH	17	17	1	8	
POSTERIORCINGULATE	LH	9			2	Activated by emotionally salient stimuli (Maddock, 1999; Maddock et al., 2003)
PRECENTRAL	LH	44	44	3	15	Activated by stimuli with emotional valence (Seo et al., 2014; Hortensius et al., 2016; Lima Portugal et al., 2020)
PRECENTRAL	RH	18		3	4	
PRECUNEUS	LH	17	17		9	Emotion regulation (Loeffler et al., 2018)
PRECUNEUS	RH	8	8		4	
ROSTRALANTERIORCINGULATE	LH	4				Emotion processing (Etkin et al., 2011)
ROSTRALANTERIORCINGULATE	RH	3				
ROSTRALMIDDLEFRONTAL	LH	18		2	3	Emotion processing (Etkin et al., 2011)
ROSTRALMIDDLEFRONTAL	RH	11			3	
SUPERIORFRONTAL	LH	43		6	7	Emotion regulation (Blair et al., 2007)
SUPERIORFRONTAL	RH	5			2	
SUPERIORPARIETAL	LH	6	6		1	Part of the attention network, necessary to carry out the task (Viviani, 2013)
SUPERIORPARIETAL	RH	10	10	2	9	
SUPERIORTEMPORAL	LH	32		1	4	When stimulated with electrical currents, may evoke emotions (Selimbeyoglu and Parvizi, 2010)
SUPERIORTEMPORAL	RH	26		2	6	
SUPRAMARGINAL	LH	36		2	15	Emotion recognition (Wada et al., 2021)
SUPRAMARGINAL	RH	33	33	3	19	
TEMPORALPOLE	LH	2				When stimulated with electrical currents, may evoke emotions (Selimbeyoglu and Parvizi, 2010)
TEMPORALPOLE	RH	6			1	
TRANSVERSETEMPORAL	LH	8			3	Auditory cortex, may be activated by inner speech while performing the task (King, 2006)
TRANSVERSETEMPORAL	RH	6			1	

The permutation column shows the number of electrode contacts pooled together to compute the permutation test, while the ML and MVPA columns show the number of electrode contacts with classification results significantly above the chance level.

### Comparison of methods for assessing condition contrasts

**FIGURE 3:**
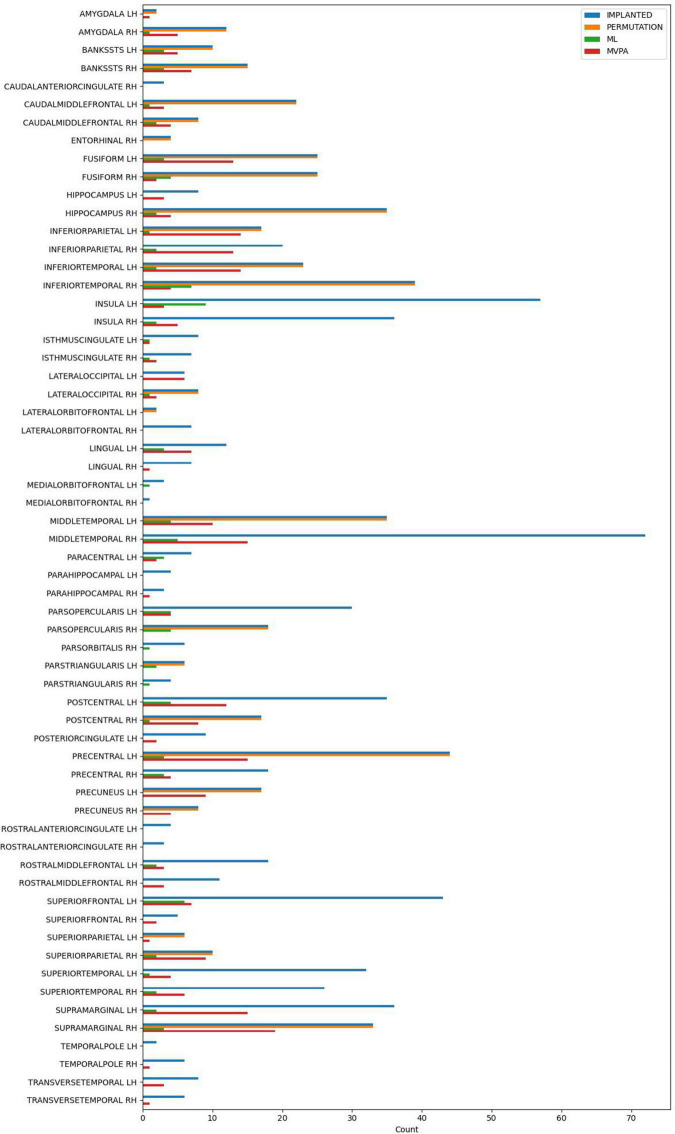
The number of implanted electrode contacts, and the number of electrode contacts exhibiting significant contrast between conditions, per brain structure and analysis method.

All the methods were compared at the same significance level of *p* < 0.05. No multiple comparison correction was used, as the *p*-value is computed using different approaches, that were described in detail above in each method’s paragraph. The number of electrode contacts deemed significant in each structure by each method is shown in Figure 3.

**FIGURE 4:**
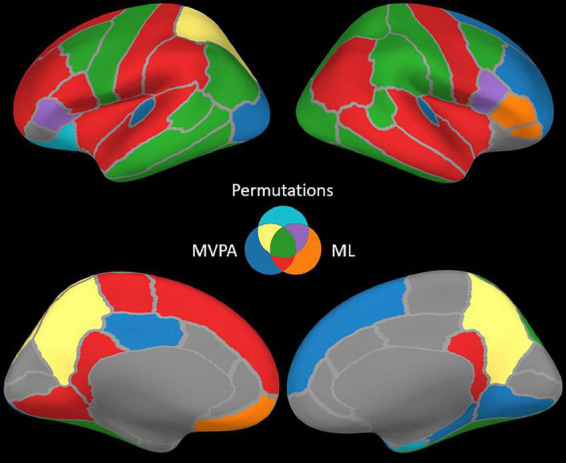
Brain structures with significant contrasts revealed by one or more methods.

The permutation cluster test identified 12 LH and 13 RH brain structures exhibiting significant contrasts between the angry and happy task conditions (Figure 4). 209 LH and 232 RH electrode contacts were grouped by structures to compute these contrasts. The two conditions resulted in significant contrasts in the bilateral amygdala and the right hippocampus, left lateral occipital cortex, parts of the parietal lobe (bilateral precuneus and superior parietal cortex, left inferior parietal cortex, and right supramarginal and postcentral gyri), parts of the temporal lobe (right entorhinal cortex and bilateral inferior temporal gyri, banks of the superior temporal sulcus and fusiform cortex, and left middle temporal), and parts of the frontal lobe (bilateral caudal middle frontal gyrus, left lateral orbitofrontal cortex, right parsopercularis, and left parstriangularis) (Table 3).

The ML classifier identified angry-happy contrasts in more brain structures (19 LH and 19 RH) than the permutation cluster test (Figure 4). Within these brain structures, a total of 55 LH and 47 RH electrode contacts exhibited an NMCC that was significantly different from the random chance. In comparison with the permutation cluster test results, the ML classifier identified significant contrasts in the bilateral superior temporal gyrus, in the right middle temporal gyrus, in the bilateral insula and the bilateral isthmus cingulate. It also identified contrasts in the following structures of the left hemisphere: lingual gyrus, superior and rostral middle frontal gyri, the parsopercularis, the postcentral gyrus, and the paracentral lobule; and the following structures of the right hemisphere: parsorbitalis, parstriangularis, and the precentral gyrus. Interestingly, several brain structures were identified by the permutation cluster test, but not by the ML classifier: the left amygdala, the left lateral orbitofrontal gyrus, the left superior parietal cortex, the right entorhinal cortex, and the bilateral precuneus (Table 3). The Jaccard coefficient computed between the structures that exhibited significant contrasts during permutation cluster tests and ML classification was 0.33 for the LH and 0.52 for the RH (Table 4).

**TABLE 4:** Pairwise Jaccard comparison between the three methods.

Comparison	Jaccard	
	LH	RH
Permutation–ML	0.33	0.52
Permutation–MVPA	0.44	0.37
ML–MVPA	0.65	0.62

The searchlight MVPA approach identified contrasts in a large number of brain structures (24 LH and 23 RH) (Figure 4), with searchlight clusters centered on 157 LH and 122 RH electrode contacts. Only the left lateral orbitofrontal cortex, right entorhinal cortex, the left parstriangularis, and the right parsopercularis were found significant by the permutation cluster test, but not by the searchlight MVPA. However, many additional structures were found significant by the searchlight MVPA, that have not been identified by either permutation cluster test or the ML classifier: the left hippocampus, the left posterior cingulate, and the left lateral occipital cortex, as well as the right lingual and parahippocampal gyri, the superior and rostral middle frontal cortices, the right temporal pole and the bilateral transverse temporal cortex (Table 3). The Jaccard coefficient between permutation cluster test results and searchlight MVPA results was 0.44 for the LH and 0.37 for the RH, and 0.65 for the LH and 0.62 for the RH between ML classifier and searchlight MVPA results (Table 4).

## Discussion

Building on the idea that humans developed a survival mechanism for immediate threat detection, it was hypothesized that two pathways are involved in the process of threat detection (LeDoux and Brown, 2017). In LeDoux’s model, the first pathway enables fast access from the retina to the amygdala, using the superior colliculus and pulvinar as relay nodes, while the second pathway is cortical and it involves the visual cortex and the fusiform gyrus. An alternative model was proposed (Pessoa and Adolphs, 2010), in which the cortex plays, through different cortical and subcortical routes, a more important role in driving visual inputs to (and back-propagated from) the amygdala through additional hubs located in the insula and orbitofrontal, frontal cingulate and posterior parietal cortices. A recent review of intracranial studies performed over the last 60 years on the topic of emotion (Guillory and Bujarski, 2014) signals the lack of intracranial data: 10 studies described the amygdala’s and 3 studies described the fusiform gyrus’ involvement in emotion processing. Moreover, only one study described the interaction between the amygdala and fusiform gyrus (Pourtois et al., 2010) at the time of the review, with a second one being published in 2016 (Méndez-Bértolo et al., 2016). Therefore, the emotion network, as described by LeDoux’s and Pessoa’s models, is understudied using intracranial methods.

The intracranial EEG studies present a specific set of challenges. The first challenge is that intracranial EEG can only be recorded from epileptic patients undergoing presurgical evaluation. In these patients, the intracranial electrodes are placed solely to localize the epileptogenic focus, therefore they may not cover all brain regions of interest for a given cognitive task, such as the facial emotion recognition task. Moreover, an emotion network as the one hypothesized by Pessoa is unlikely to be fully observed with intracranial electrodes for two reasons: (1) the network extends on multiple lobes, while the intracranial implantation schemes are usually focused on 1–2 lobes, and (2) the subcortical nuclei, such as the pulvinar, are not common targets for presurgical evaluation for epilepsy. Therefore, the emotion networks are prone to be studied at the group level, to overcome the brain spatial sampling issues. A second challenge relates to the effect size that is to be observed as the contrast between two conditions. It was estimated that such contrast can be as low as 3% of the signal-to-noise ratio (Selimbeyoglu and Parvizi, 2010). A third challenge is represented by the fact that there is no ground truth for what is the brain network activated by the facial emotion recognition task. In our study, we leverage previous studies and theoretical models (Pessoa and Adolphs, 2010) to explain why one of the three methods might highlight contrasts in a given brain structure. This approach is, however, a rough approximation, as most relevant studies were performed using fMRI, and the intracranial EEG literature on emotion processing is still limited (Guillory and Bujarski, 2014).

The permutation cluster test showed a larger number of contrasts in the RH than in the LH, a finding that is consistent with the “Right Hemisphere” model for emotional processing (Silberman and Weingartner, 1986; Demaree et al., 2005). Of the LH contrasts observed, the amygdala and the fusiform gyrus, the inferior and superior parietal and the lateral orbitofrontal, and the caudal middle frontal cortex are worth mentioning as they partially outline the non-occipital parts of the Pessoa model. In addition, we observed contrasts that are likely related to the task execution: decision-making in the lateral orbitofrontal and the caudal middle frontal cortex (Talati and Hirsch, 2005; Nogueira et al., 2017) and movement execution for the button-press in the precentral gyrus (Li et al., 2015). The same network was also observed in the RH, with additional contrasts in the hippocampus and entorhinal cortex [structures associated with the encoding and recognition of facial expressions (Fried et al., 1997)], the parsopercularis and the supramarginal gyrus, which takes part in the perception (Belyk et al., 2017) and recognition of emotion (Wada et al., 2021), respectively.

Moving from the permutation cluster test to the univariate (ML) and the multivariate (searchlight MVPA) classification methods, we observed an increase in the number of brain structures with significant contrasts (Figure 4 for qualitative results and Table 3 for the exact number of intracranial electrode contacts per brain structure). While some of these are part of Pessoa’s model, like the insula and the cingulate gyrus, other structures are surprising and not commonly associated with the processing of faces and emotions. However, these findings support the idea of larger and distributed networks for emotion processing, which encode the different types of emotions as activation patterns (Wager et al., 2015). The generalization power of classification methods is, in our view superior to the statistical method of permutation cluster test (Figure 4), as it can be demonstrated to classify trial conditions with unseen data (Cauchoix et al., 2014; Kragel and LaBar, 2016). However, while the participation of such brain structures in the realization of the cognitive task is undeniable, it is still debatable if they are part of the core emotion processing network, or if they participate in more general aspects of the task such as low-level visual processing, movement planning, inner speech evoked unconsciously by the images, etc. The banks of the superior temporal sulcus, which appeared significant for both ML and searchlight MVPA bilaterally, are considered an integration hub for audiovisual stimuli, inner speech, motion, and face processing (Hein and Knight, 2008). The Jaccard index showed a good agreement (∼0.65) between the ML and MVPA methods on both hemispheres, which is expected as both methods rely on different, yet similar, machine learning classifiers, and the input data are overlapping. A lower Jaccard index value (∼0.42) is observed between the permutation cluster test and each other method on the RH. Therefore, the searchlight MVPA approach integrates information from a larger brain volume, resulting in larger Jaccard index when compared to the permutation cluster test.

Several other fMRI studies have reported widely distributed brain regions that encode recognition of different emotional states through patterns of activations that were identified using MVPA (Kassam et al., 2013; Kragel and LaBar, 2016). Despite MVPA’s popularity in the fMRI field and its applicability to scalp EEG (Cauchoix et al., 2014) to our knowledge, only four studies leveraged MVPA techniques with intracranial EEG to study rapid visual categorization in the ventral stream in rhesus macaques (Cauchoix et al., 2016), fast visual recognition memory systems (Despouy et al., 2020), and semantic coding in humans (Chen et al., 2016; Rogers et al., 2021), to date. The searchlight MVPA method we implemented in this study allowed us to explore for the first time arbitrary spherical brain volumes centered on an intracranial depth electrode contact. Despite the variable number of electrode contacts contributing to this analysis at the patient level, we observed that the electrode contacts exhibiting statistically significant accuracies above the chance level tend to cluster at the group level.

Multivariate pattern analysis and its searchlight implementation appear to be more sensitive to small differences between conditions and therefore reveal a widespread brain network involved in emotion processing, which contradicts the ‘standard hypothesis’ model that considers the emotion network to have a cortical and subcortical route that connects the visual cortex to the amygdala (Pessoa and Adolphs, 2010), but supports the “multiple waves” model (Pessoa and Adolphs, 2010). A wider brain network provides more opportunities to modulate the brain functions, that have previously focused on the amygdala stimulation (Inman et al., 2018), and may explain the success of emotion regulation of various transcranial magnetic stimulation studies that focused on the prefrontal cortex (De Wit et al., 2015; Lantrip et al., 2017), a brain region that is part of the “multiple waves” model and was found to play an active role in the processing of different types of emotions as part of a widespread brain network (Wager et al., 2015).

We observed that the permutation cluster test has identified the least number of brain structures exhibiting a significant contrast between task conditions, followed by the ML classifier and then the searchlight MVPA. This behavior is expected and explained by the particularities of each method. The permutation cluster test is appropriate to detect large differences (clusters), but has a low sensitivity for small clusters that are commonly observed in EEG data (Nichols and Holmes, 2002; Huang and Zhang, 2017). The machine learning classifier can have better sensitivity, as even a single feature that is systematically different between the two task conditions is enough to provide good classification performance. In our study, we computed features over 200 and 400 ms intervals, but the five features we have used describe the data underlying these intervals sufficiently well to identify more brain structures with significant task contrasts than the permutation cluster test. The searchlight MVPA has all the benefits of the ML classifier, and adds the spatial dimension to the analysis: it considers the multi-variate changes in the features computed for all intracranial contacts within a predefined search radius. As expected, the results of searchlight MVPA were the best, this method identifying the largest number of brain structures with significant task condition contrasts, all of them being in agreement with the existing scientific literature on face recognition and emotion processing (Table 3).

## Conclusion

This manuscript provides the first methodological side-by-side comparison of three methods for identifying task contrasts in EEG data and exemplifies the usage of searchlight MVPA with intracranial depth electrodes. However, an in-depth analysis of the brain networks identified by searchlight MVPA and the neuroscientific interpretation of the findings is beyond the scope of the current study, which is only aimed at validating the results through the existing literature. We have shown that the permutation cluster analysis, which is commonly used for the analysis of intracranial EEG data, is less sensitive to task contrasts than ML classification, and both of them are less sensitive than searchlight MVPA method. Of course, none of these three methods has identified significant task-related contrasts in all brain structures featured in Table 3, even though each of those structures is involved in one way or another in the processing of faces or emotions, according to the previous studies we have referenced.

At the same time, our study is the first intracranial EGG study to reinforce the idea that the emotion network is widespread and relies on activation patterns to process various emotions, as demonstrated by an fMRI study (Kragel and LaBar, 2016) and a meta-analysis of 148 emotion-related studies (Wager et al., 2015).

A detailed analysis of the searchlight MVPA brain networks and their temporal dynamics through time generalization (King and Dehaene, 2014; Rogers et al., 2021) will be addressed in a future study.

## Data availability statement

The raw data supporting the conclusions of this article will be made available by the authors, without undue reservation.

## Ethics statement

The studies involving human participants were reviewed and approved by Ethical Committee of University of Bucharest, approval 43/02.10.2019. The patients/participants provided their written informed consent to participate in this study.

## Author contributions

CD: conceptualization, methodology, validation, software, formal analysis, writing—original draft, visualization, supervision, and funding acquisition. BB: software. CP: software and data curation. IO: data curation. IM: validation, writing—review and editing, and supervision. AB: validation, writing—review and editing, supervision, and funding acquisition. All authors contributed to the article and approved the submitted version.

## Abbreviations

ML: machine learning
MVPA: multivariate pattern analysis
fMRI: functional magnetic resonance imaging.
